# Placement matters: Implications of trail‐ versus random‐based camera‐trap deployment for monitoring mammal communities

**DOI:** 10.1002/eap.70083

**Published:** 2025-08-07

**Authors:** Ilaria Greco, Marco Salvatori, Elena Buonafede, Alessandra Pistolesi, Andrea Corradini, Nadia Cappai, Matilde Marconi, Lorenzo Seidenari, Francesca Cagnacci, Francesco Rovero

**Affiliations:** ^1^ Department of Biology University of Florence Sesto Fiorentino Italy; ^2^ MUSE – Museo delle Scienze Trento Italy; ^3^ Animal Ecology Unit Fondazione Edmund Mach, Research and Innovation Centre San Michele all'Adige Italy; ^4^ National Biodiversity Future Center (NBFC) Palermo Italy; ^5^ Foreste Casentinesi National Park Pratovecchio‐Stia Italy; ^6^ Department of Information Engineering University of Firenze Florence Italy

**Keywords:** anthropogenic disturbance, biodiversity monitoring, camera‐trapping protocols, mammal communities, sampling design

## Abstract

Optimizing protocols to assess and monitor mammal communities is essential to meet the current biodiversity targets of halting species loss. Camera‐traps are the most effective tool for multispecies monitoring, yet their deployment strategy is debated, with two main strategies adopted: trail‐ and random‐based camera deployment. To date, few studies have compared these two strategies and reached contrasting recommendations. Here, by simultaneously deploying 60 camera‐traps for each placement strategy in a National Park in central Italy, we aimed to assess differences in species richness and composition, photographic rate, detection/occupancy probabilities, also in responses to environmental and anthropogenic variables, and temporal activity. Site species richness was greater on than off‐trails, with elusive carnivores mainly detected on trails. Community composition was different, with a smaller proportion of ungulates on trails, and lower detections of carnivores off‐trails. Photographic rate, detection, and occupancy probabilities were higher on trails for almost all mammals. Occupancy responses to environmental variables did not match, possibly due to the different behavioral strategy adopted by mammals (trails for movement, off‐trails for resting and foraging). Thus, a mixed approach with cameras located both on‐ and off‐trails is recommended when studying habitat use. We also found a consistent negative response of occupancy and site‐use intensity to human frequentation, with mammals avoiding both highly frequented trails and adjacent random sites. Temporal activity curves were similar between designs, suggesting that the choice of the sampling strategy would not bias the inference. However, nocturnal behavior was higher on trails for some species, indicating varying degrees of temporal avoidance of humans. With faster data accumulation, easier accessibility of sampling sites, and the ability to record human activity, on‐trail cameras are more efficient than off‐trail cameras for monitoring mammal communities.

## INTRODUCTION

Conservation and management practices should be based on scientifically sound assessments, able to gauge the status of wildlife and its response to sources of disturbance. Robust data from standardized monitoring programs are the only way to assess populations and communities through time reliably, and to quantify the efficacy of conservation and management strategies (Sutherland et al., 2019). Monitoring is fundamental to assess anthropogenic impacts on biodiversity and set conservation priority goals (Mihoub et al., 2017; Robinson et al., 2018). Recently, the monitoring paradigm has been shifting from surveying target species to communities (Chaudhary et al., 2022), for the higher ecological implications at the ecosystem level (Jetz et al., 2019), and as a cost‐effective practice to collect information on multiple species in the face of limited resource availability (Kunming‐Montreal Global biodiversity framework, 2022).

Camera‐traps are one of the most effective tools for monitoring mammal communities across habitats, latitudes, and spatiotemporal scales (Bruce et al., 2025; Wearn & Glover‐Kapfer, 2019), with a high potential for standardization enabling data comparisons in time and space (Steenweg et al., 2017). This technology has played a pivotal role in the transition from single‐ to multispecies research (Burton et al., 2015): their capacity to detect any passing wildlife in front of the camera lens allows recording the full array of medium‐to‐large wild mammals with a single technique (Hofmeester et al., 2017).

However, the wide application of camera‐trapping has implied the use of a disparate set of sampling designs, and the relative efficacy of these designs is still unclear. Studies that evaluated the implications of various sampling strategies and efforts (Beaudrot et al., 2019; Kays et al., 2020; Rovero et al., 2013) generally recommend the systematic deployment of cameras according to a regular grid with a fixed density of cameras, allowing sampling of large areas evenly, and better capturing the diversity of whole communities of medium‐to‐large mammals (Jansen et al., 2014; Rovero et al., 2013; Wearn & Glover‐Kapfer, 2017).

Systematic camera‐trapping can however derive from different sampling designs, especially regarding the choice of sampling sites. Two alternatives are usually employed (Meek et al., 2014): (1) targeted sampling, with remote devices targeting specific features such as roads, game/hiking trails, or water sources (Cusack et al., 2015); and (2) random, not targeting specific features. Targeted deployments (hereafter on‐trails), particularly roads/trails‐oriented, are routinely used in wildlife research, already providing standardized data for large‐scale monitoring (Cove et al., 2021; Rovero & Ahumada, 2017).

A major strength is the increased likelihood of capturing carnivores (Blake & Mosquera, 2014; Fonteyn et al., 2021) for their use of trails and roads as travel routes. However, results could be biased toward species that preferably use such features, limiting their generalization to the whole community (Anderson, 2001). Different species can indeed use the trail network with various intensities (Harmsen et al., 2010), making some species more detectable than others. Particularly, some mammals avoid these features (Blake & Mosquera, 2014) to limit encounters with predators or humans (Harmsen et al., 2010; Weckel et al., 2006). Placement on preferential travel routes can also introduce bias toward linear movement behaviors, underrepresenting behaviors as foraging, searching or resting (Caravaggi et al., 2018).

Random placements (hereafter off‐trails) overcome the aforementioned biases since any environmental feature would be presumably sampled in proportion to its occurrence in the landscape, and different animal behaviors would have the same probability of being detected (Palencia et al., 2019; Rowcliffe et al., 2013). Furthermore, off‐trail deployments are required to estimate densities of unmarked animals using approaches such as the Random Encounter Model (Rowcliffe et al., 2008) or Distance Sampling (Howe et al., 2017). Yet, fully randomized designs are rarely applied in practice (Burton et al., 2015), due to the logistically demanding data collection and the severely lower number of photographs (Sollmann et al., 2013).

Understanding the benefits, drawbacks, and implications for ecological inference of these two sampling strategies can aid in choosing the most appropriate design when starting a new monitoring program, especially when the whole community of medium‐to‐large mammals is the target. Species in the community might be detected differently with alternate sampling designs, potentially leading to unpredicted bias and confounding community‐level results (Iannarilli et al., 2021).

To date, few studies have assessed the effectiveness and performance of different camera‐based monitoring strategies, and even fewer have focused specifically on comparing off‐ versus on‐trail deployment. The recommendations from the pool of available studies are seemingly contrasting (Appendix S1: Table S1): Cusack et al. (2015), Kolowski and Forrester (2017) and Wearn et al. (2013) advocate for the use of a off‐trail placement for unbiased estimations. Tanwar et al. (2021) support the use of trail‐based design, although acknowledging a less rigorous inference for herbivores. Blake and Mosquera (2014), Di Bitetti et al. (2014), Hofmeester et al. (2021), and Iannarilli et al. (2021) suggested a mixed approach for a more comprehensive view of the mammal community, while Fonteyn et al. (2021), like Cusack et al. (2015), found that camera placement did not critically affect monitoring outcomes. Notably, a comparison of estimates of occupancy from off‐ versus on‐trail sampling has not been performed, despite the wide use of this metric in camera‐trapping studies; similarly, it has not been assessed whether data from the two designs assess associations with environmental and anthropogenic variables in similar or different ways.

Here, by simultaneously using systematic off‐ and on‐trail designs, each deploying 60 camera sites in the same study area (a temperate forest in central Italy), we aimed to assess the benefits and drawbacks of the two sampling protocols to monitor mammal communities. We compared the two sampling designs in terms of: (1) species richness and composition, (2) camera‐trap photographic rate (Rovero & Marshall, 2009), (3) detection and occupancy probabilities, (4) species responses to environmental and anthropogenic factors in terms of variation in occupancy and site‐use intensity, and (5) temporal activity. We predict that species richness is higher on‐ than off‐trails, with a faster species accumulation rate and elusive species only recorded along trails; species composition is different, with a greater proportion of carnivores detected from trail‐based cameras; species‐specific photographic rates, detection, and occupancy are lower at off‐trail sites for all species; species‐specific occupancy varies similarly, between designs, in relation to environmental factors; wild mammals are more diurnal at off‐trail cameras and nocturnal along trails and forestry roads due to greater anthropogenic disturbance.

Furthermore, the simultaneous use of cameras on‐ and off‐trails offers the unparalleled opportunity to test if mammals' space‐use is affected by human disturbance even when animals are located outside the network of forestry roads and trails. Hence, by using the Cumulative Outdoor activity Index (COI; Corradini et al., 2021), a metric based on human mobility data, we evaluated if the use of the landscape by humans has potential detrimental effects on wild mammals affecting their space‐use. We predicted that the perceived disturbance determined by human presence penetrates beyond forestry roads and trails, with animals avoiding off‐trail sites closer to highly frequented linear features. Lastly, by calculating costs and personnel required to deploy each sampling strategy, we compared the cost‐effectiveness of the two protocols.

## MATERIALS AND METHODS

### Study area

**FIGURE 1:**
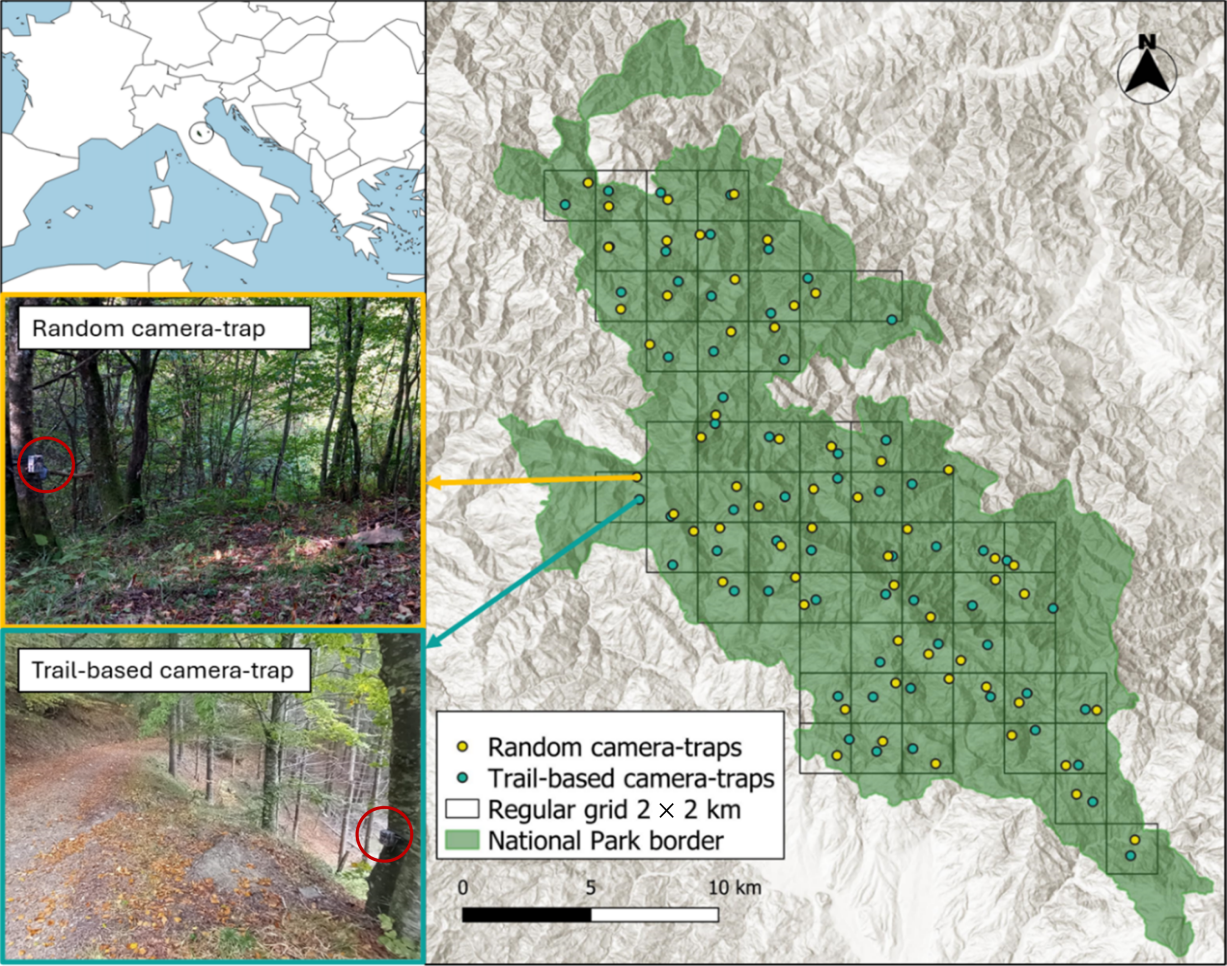
Map of the study area (Parco Nazionale Foreste Casentinesi) across which the regular grid has been developed. The main figure shows the paired distribution of the trail‐based (light blue) and randomized (yellow) camera‐traps. Insert maps report the location of the study area in central Italy (top left), and examples of a camera‐trap deployed randomly (yellow‐bordered insert figure) and a camera‐trap located along a forestry road (light blue‐bordered insert figure). Data were collected between August and November 2023. Camera‐trap photograph credits: Ilaria Greco.

The study took place in Parco Nazionale Foreste Casentinesi (PNFC, 43°480 N, 11°490 E), a protected area of 36,426 ha gazetted in 1993, and located on the Apennine ridge of central Italy (Figure 1). The park has an altitudinal range spanning from about 400–1658 m asl at the highest peak. The climate is temperate—sub‐Mediterranean, characterized by cool and humid summers and relatively cold winters with occasional snowfalls (Viciani et al., 2010). PNFC has one of the highest national forestry coverages: 80% of the park's surface is covered with dense forests, with beech (*Fagus sylvatica*) and Turkey oak (*Quercus cerris*) as dominant tree species (www.parcoforestecasentinesi.it). PNFC presents an extensive network of forestry roads and hiking trails, and 12 municipalities are located inside its border. With 2000 permanent residents (www.parcoforestecasentinesi.it), human density is relatively low (ca. 5.5 ind./km^2^), although human frequentation strongly increases during summer for outdoor recreation.

PNFC hosts a rich mammal community of 14 medium‐to‐large species (Salvatori et al., 2024), among which there are four ungulate species (i.e., red deer *Cervus elaphus*, roe deer *Capreolus capreolus*, fallow deer, *Dama dama* and wild boar *Sus scrofa*), 13 detected wolf (*Canis lupus*) packs (Dissegna et al., 2023), a population of wild cat (*Felis silvestris*; Anile et al., 2025), and an invasive population of raccoon (*Procyon lotor*) first recorded in 2013 (Boscherini et al., 2020). This latter species is of management concern given it represents the main cause for the population decline of the native white‐clawed crayfish (*Austropotamobius pallipes*) in PNFC (Tricarico et al., 2021).

### Sampling designs, data collection, and management

Data collection was carried out between August and November 2023, with the simultaneous installation of both on‐ and off‐trail camera‐traps. Both sampling designs were based upon a regular grid of 2 × 2 km cell size laid across the whole territory of the park (Figure 1). The grid consisted of 60 sampled cells within which one camera located on a forestry road or a trail and one randomly located camera were deployed (Figure 1). On‐trail sampling sites were located to the closest accessible trail or forestry road to the centroid of each cell, facing the trails at 2–3 m from their center. Off‐trail sites were randomly generated within each cell with the dedicated built‐in function in Quantum GIS 3.16.11 (QGIS Development Team, 2019) and framed a relatively homogeneous area free from ground vegetation. Once in the field, we tried to locate the off‐trail site as close as possible to the theoretical point, with minor adjustments due to accessibility or topographic constraints.

Camera‐traps were 24 h‐active in the field for at least 30 consecutive days and were attached to tree trunks at approximately 50–60 cm. Due to equipment constraints and for logistic optimization, we divided the grid into two sub‐grids of 30 sites sampled sequentially, each for 30 days. We employed Browning Dark Ops (Birmingham, AL, USA) cameras, set to work in continuous mode and to produce rapid‐fire sequences of eight photographs (shots belonging to the same sequence had a 0.2 s delay) with a delay of 1 s between sequences.

After camera retrieval, photographs were processed through a dedicated software (Wild.AI) hosted on a platform by the University of Florence that aids in the storage, management, and identification of photos. This latter process was carried out through an in‐built detector that classifies photos into four major categories: “Blank,” “Humans,” “Vehicles” and “Wildlife.” Photos belonging to the “Wildlife” category were then manually classified to species level by three authors (i.e., Ilaria Greco, Elena Buonafede, and Alessandra Pistolesi), who each independently annotated the images from 40 sampling sites (20 on‐trail and 20 off‐trail). In cases of doubtful identifications, determined when the confidence in species identification was ranked as low, the images were shared with the other two colleagues for confirmation. Consensus was generally reached except for 17 images whose subjects were too blurred. These images, all belonging to the off‐trail sampling, were excluded from the analyses. On the whole, only 17 photographs from the off‐trail design had this label.

## DATA ANALYSES

### Species richness, composition, and photographic rate

We calculated randomized species richness accumulation curves for both sampling designs following Gotelli and Colwell (2001), with 1000 random permutations, to visualize the effectiveness of the two sampling strategies at detecting species of the medium‐to‐large mammalian community. We also compared on‐ and off‐trail site‐level species richness and tested for differences using pairwise Wilcoxon signed‐rank tests. We then compared community composition based on species' relative abundance resulting from the two designs using multivariate techniques. The multivariate distances among sites were estimated with a Bray–Curtis Dissimilarity Index, and the resulting distance matrix was then analyzed through a nonmetric multidimensional scaling (nMDS) ordination analysis using the function metaMDS in the R (R Core Team, 2022) package *Vegan* (Oksanen et al., 2015) on the resulting distance matrix.

Relative abundances of each species (i.e., photographic rate) were quantified by the relative abundance index (RAI), that is, the number of independent photographic events of each species over the effort (number of sampling days) and multiplied by 100. Photos of the same species at the same camera location were considered independent when taken >30 min apart (Rovero & Spitale, 2016). Differences in species composition were then tested with a permutation‐based multivariate ANOVA (npMANOVA), with 999 permutations. Lastly, differences in the species' RAIs between sampling designs for each species were also tested using pairwise Wilcoxon signed‐rank tests.

### Multispecies models of occupancy and site‐use intensity

To assess if species‐specific detection probability (*p*), occupancy (ψ) and their response to specific environmental variables were affected by camera deployment strategies, we used two separate multispecies occupancy models (MSOMs; Kéry & Royle, 2015). The wolf (*N* = 5) and the wild cat (*N* = 5) for the off‐trail design and the raccoon (*N* = 1) for the trail design were discarded for the low number of independent detections.

Species‐specific detection probabilities were modeled as a function of the distance to the closest municipality, the mass of detected mammals to correct for the potentially higher detection of larger‐sized species, and, for the trail‐based design only, a categorical variable indicating whether a camera was installed on a forestry road or a trail. Species‐specific occurrence probabilities were modeled as a function of elevation, terrain slope, distance to the closest municipality, distance to the park's border, and Cumulative Outdoor activity Index (COI). This latter index measures the intensity of use of trails by people during leisure activities, representing an approximation of the intensity of potential human disturbance (Corradini et al., 2021). We validated the COI index by calculating the Spearman correlation coefficient against the photographic rate of humans obtained by trail‐based cameras to evaluate its reliability as an index of human passage in the study area.

Moreover, we also separately modeled species‐specific detection events, taken as a proxy of site‐use intensity, with the same set of independent variables used for occupancy modeling. See Appendix S1: Supplementary text “Specifications for the Multi‐Species Occupancy Model and Generalized Linear Models on site‐use intensity” for model specification and covariates description.

### Temporal activity

Differences in species‐specific temporal activity patterns between on‐ and off‐trail camera traps were estimated through Kernel density distributions from the time stamps of independent events, by using the functions implemented in the R package *Overlap* (Meredith & Ridout, 2021). We considered only species captured with both sampling designs that had a minimum of 10 independent events. Then, we assessed the degree of overlap between pairs of curves belonging to the same species from both designs by using the overlap coefficient Δ, that ranges from 0 (no overlap) to 1 (complete overlap) using the R package *Activity* (Rowcliffe et al., 2014). We chose Δ_1_ (*N* < 50) or Δ_4_ (*N* > 50) according to the sample size (Meredith & Ridout, 2021). The 95% CIs for the overlap coefficients were estimated by computing 1000 bootstrap iterations (Ridout & Linkie, 2009).

Lastly, we tested for significant differences between the two pairs of curves with a Wald test (Greco et al., 2022). Additionally, following Procko et al. (2023), we also assessed potential on‐ and off‐trail differences in temporal activity in relation to the peak of human disturbance (i.e., midday) calculating for each species‐specific detection event the time between the event and solar noon. This is a continuous metric that goes from 0 (midday) to 12 (midnight) hours and measures the tendency toward nocturnality as a temporal distance from the peak of human activity (e.g., a value of four represents a photographic event taken at 4 p.m. or at 8 a.m., therefore 4 h away from solar noon), with higher values indicating more nocturnal events. We then assessed differences in nocturnality between the two sampling designs through a Wilcoxon rank sum test for each species.

## RESULTS

### Species richness, composition, and photographic rate

We obtained data from a total of 118 camera‐traps, as 59 for each sampling strategy worked effectively. Sampling effort for the off‐trail protocol reached 1705 camera‐trapping days (28.9 mean days per camera) and 1794 (30.4 mean days) for the trail‐based protocol. While costs for the off‐ and the on‐trail monitoring project were fairly equivalent, we calculated that the effort needed for the off‐trail design was 1.5 times greater than the trail‐based one (37 vs. 24 person‐days, respectively; Appendix S1: Figure S1).

**FIGURE 2:**
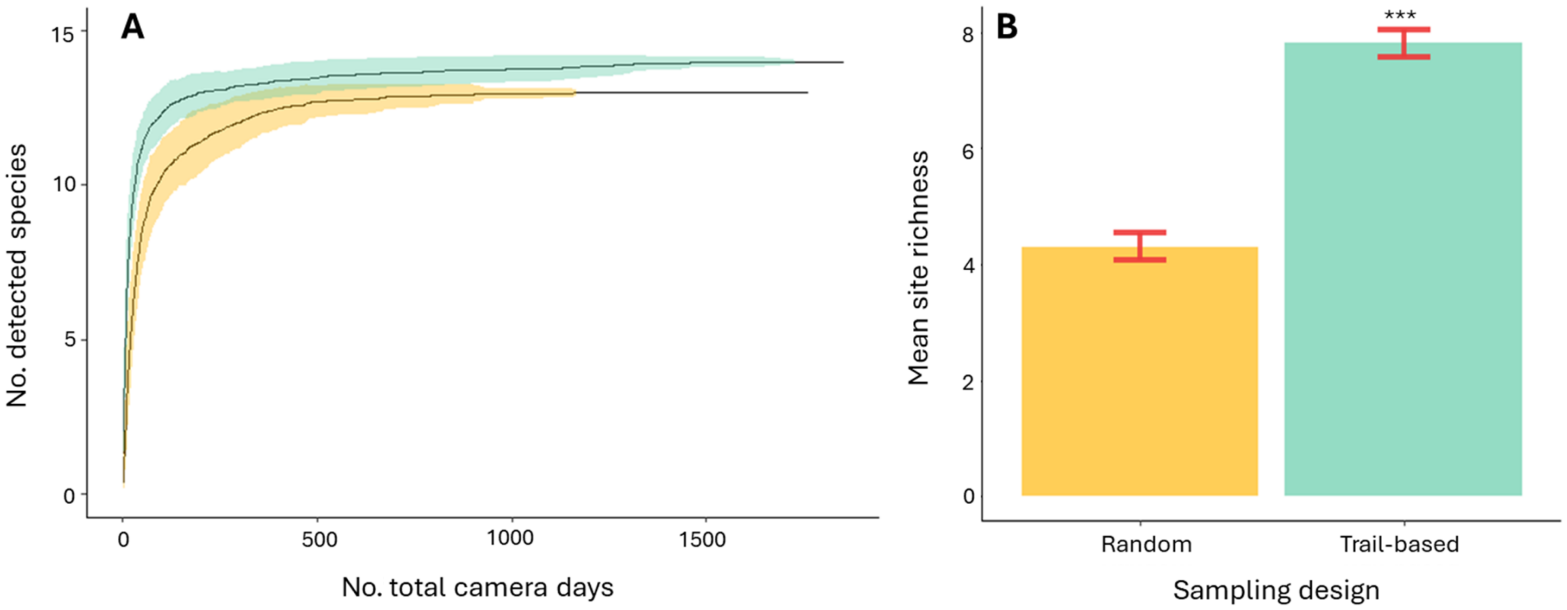
(A) Species accumulation curves estimated for the off‐trails (yellow) and the on‐trail design (light blue). (B) Average site‐level richness for the two designs. Asterisks represent significance level at *p* < 0.001.﻿﻿﻿﻿﻿﻿

Both accumulation curves reached a plateau (Figure 2A), attesting to the effectiveness of both monitoring strategies, although with a slightly faster accumulation rate along trails. Thirteen species of medium‐to‐large mammals were detected with the off‐trail protocol and 14 on the trail‐based one, with the raccoon only recorded on the latter. Site‐specific richness of wild mammals was significantly higher on than off trails (*v* = 1467.5, *p* < 0.001, Figure 2B), with a mean of eight species (±0.23 SD) recorded on the trail network and 4 species (±0.24 SD) recorded on off‐trail sites, confirming the prediction of higher species richness and faster accumulation on trails.

**FIGURE 3:**
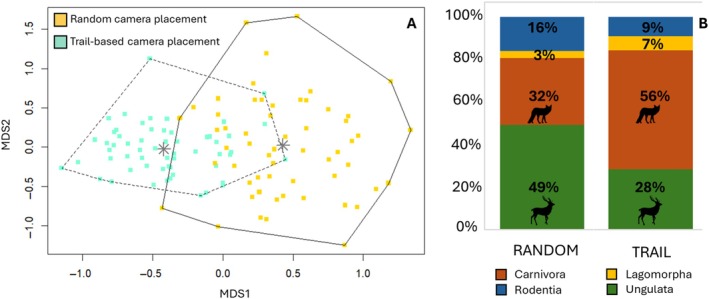
(A) Nonmetric multidimensional scaling plot (nMDS) of medium‐to‐large mammal species composition according to the species relative abundances recorded with the off‐trails (yellow) and the on‐trail (green) camera‐trapping design. Polygons highlight clusters based on sampling designs with gray asterisks representing their centroids (i.e., average position of observations in ordination space). (B) Species composition according to the proportion of relative abundance of the four main mammal orders estimated from camera‐trapping data collected with the off‐trails (left‐wing side) and the on‐trail sampling design (right‐wing side). Wildlife silhouettes were downloaded from the public domain website Openclipart (https://openclipart.org/).

When considering all the taxa detected (Appendix S1: Table S2), we obtained 6‐times more data on‐ than off‐trails (Appendix S1: Figure S2), with 52% of independent events depicting wild mammals, 39% people and 9% domestic species (i.e., cats, dogs, and livestock). Conversely, 94% of the independent events derived from the off‐trail design represented wild mammals, and only 5% people and 1% domestic species (Appendix S1: Figure S2). Species composition in terms of relative abundances was significantly different between the two sampling designs (*F*_1,116_ = 18.11, *p* < 0.001), as also illustrated by the 2D nMDS ordination plot (Figure 3A; stress = 0.17). The differences in community composition between the two sampling designs were apparent when considering the RAI aggregated by taxonomic group: wild ungulates represented 49% of the total RAI for the off‐trail sampling versus 28% on the on‐trail sampling. Rodents (i.e., crested porcupine *Hystrix cristata* and red squirrel *Sciurus vulgaris*) had a similar trend (16% vs. 9% for off‐ and on‐trails, respectively). Conversely, carnivores and lagomorphs had an opposite pattern, with 32% versus 56% for carnivores and 3% versus 7% for lagomorphs for off‐ and on‐trails, respectively (Figure 3B). The prediction of different community compositions from the two designs was therefore confirmed.

**FIGURE 4:**
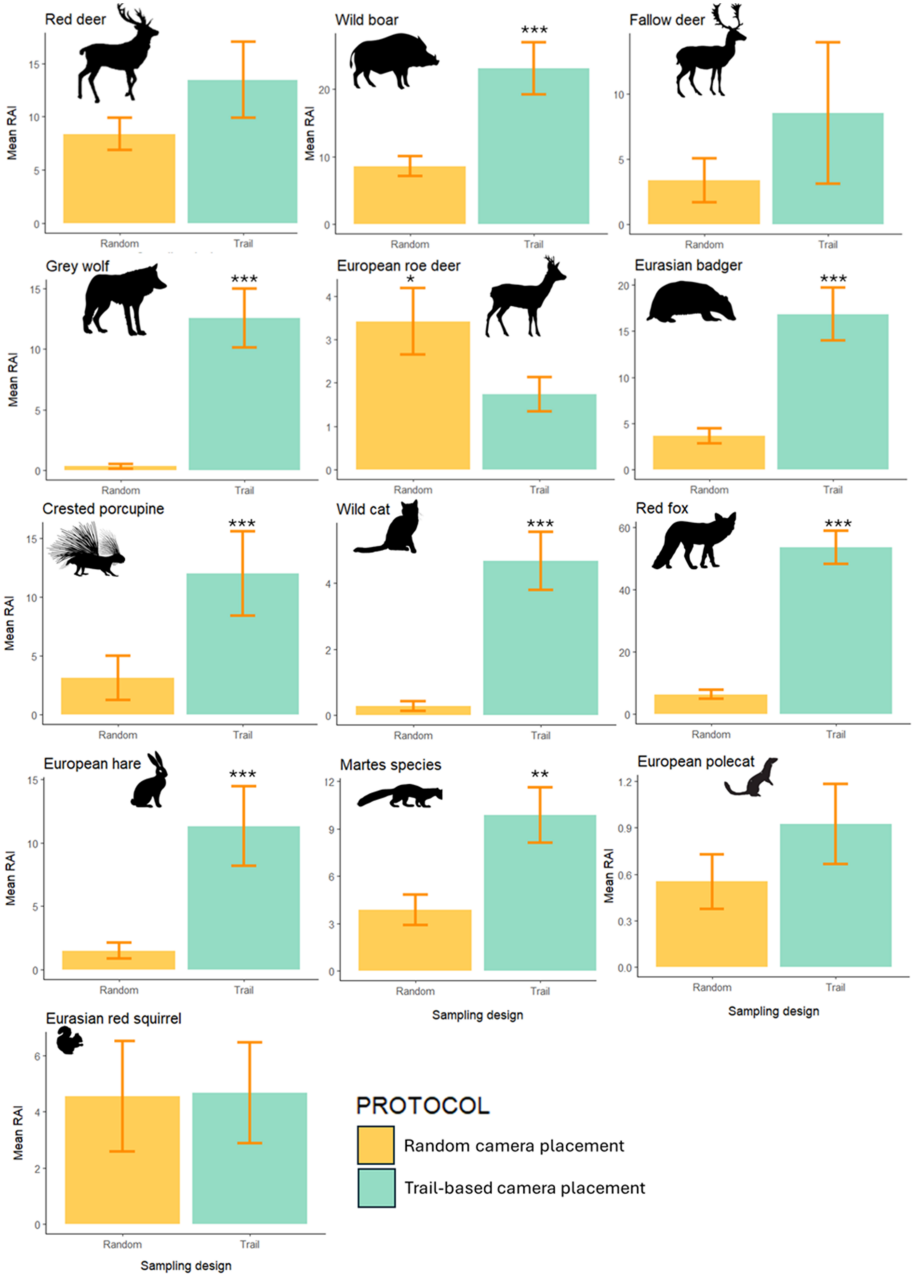
Comparison between species‐specific photographic rate (RAI—relative abundance index) derived from the off‐trails (yellow) and the on‐trail (light blue) sampling design. Species are ordered according to decreasing body mass. Asterisks indicate significant differences estimated with a pairwise Wilcoxon signed‐rank test (*p < 0.05; **p < 0.01; ***p < 0.001). Wildlife silhouettes were downloaded from the public domain website Openclipart (https://openclipart.org/).

Photographic rate (RAI) was significantly higher on trails for almost all detected species (Figure 3), except for the red deer (*v* = 838, *p* = 0.57), fallow deer (*v* = 301, *p* = 0.49) and polecat *Mustela putorius* (*v* = 92, *p* = 0.92), for which the difference was not significant. The squirrel (*v* = 1985, *p* = 0.69) had a comparable photographic rate between on‐ and off‐trails, while the roe deer (*v* = 199, *p* = 0.02) was the only species with a significantly greater photographic rate at off‐trail sites (Figure 4). Two carnivores, the wolf and the wild cat, yielded only five independent events each off‐trails, compared to 242 and 80 events on trails for the wolf and wild cat, respectively (Appendix S1: Table S2; Figure 4).

### Multispecies models of occupancy and site‐use intensity

Eight out of the 11 species tested had higher detection and occupancy on‐ than off‐trails (Appendix S1: Figure S3). In line with their results for photographic rate, wild boar, badger (*Meles meles*), crested porcupine, fox, *Martes* spp., and hare had higher detection and occupancy on‐ than off‐trails. Red deer, which did not have a significantly different photographic rate (Figure 2) had instead higher detection and occupancy on trails (Red deer: *p*, *v* = 1586, *p* < 0.001; ψ, *v* = 1230, *p* = 0.013). Polecat and squirrel had similar estimated detection and occupancy across the study area with the two protocols (Appendix S1: Figure S3), while roe deer (*v* = 112; *p* < 0.001) and fallow deer (*v* = 127, *p* < 0.001) were the only species with a significantly greater detection probability off‐ than on‐trails, although the difference in roe deer occupancy was not significant (*v* = 655; *p* = 0.08) and favored trails for fallow deer (*v* = 1360; *p* < 0.001). The prediction of lower photographic rate, occupancy, and detectability off‐ than on‐trails was generally confirmed, with the exception of red squirrel (no difference) and roe deer (higher values off‐trails than on‐trails). For both protocols, we found weak support that greater body mass positively influenced mammal detections (Appendix S1: Table S3).

**FIGURE 5:**
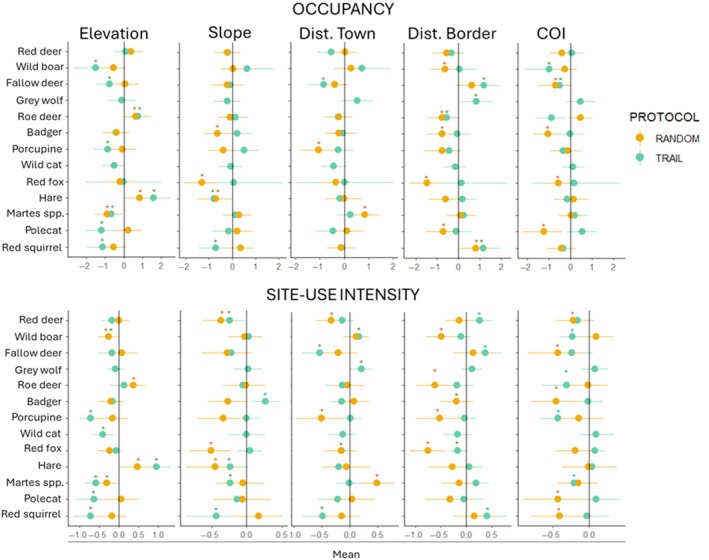
Coefficient estimates for the occupancy probability (above) and the site‐use intensity—number of independent detection events—(below) for all medium‐to‐large mammal species detected with off‐trails (yellow) and on‐trail monitoring (light blue) protocol. Values represent the mean of the site‐specific posterior distributions with 90% Bayesian credible intervals (BCI). Asterisks highlight when 90% BCI does not overlap 0. COI, Cumulative Outdoor activity Index.

Modeling occupancy and site‐use intensity in dependence on environmental and anthropogenic covariates showed that coefficient signs were concordant 73 times out of 110 (66.36%) between the coefficient estimates from two sampling designs (Figure 5), partially confirming the prediction of similar environmental associations with data from the two sampling designs. Four main possibilities emerged: (1) in 11.82% of cases, the two regression coefficients from the two sampling designs had the same signs and were both significantly different from 0; (2) in 27.27% of cases, the regression coefficients for the same variable were significant for only one of the two sampling designs, though they had the same sign and their credible intervals largely overlapped; (3) in 24.82% of cases, the two coefficients had the same signs but both had 90% CI that overlapped 0; (4) in 33.64% of cases, the two coefficients for the same variable had different signs, but they were both significantly different from 0.

COI was positively correlated with the on‐trail photographic rate of humans, with a Spearman's correlation coefficient *R* = 0.45 (*p* < 0.001; Appendix S1: Figure S4). In general, 10 out of 13 species (i.e., red deer, wild boar, fallow deer, roe deer, badger, porcupine, red fox, hare, polecat, and red squirrel) showed significantly negative responses to human frequentation on either occupancy or site‐use intensity. This result was more evident for the off‐ than on‐trail sampling (nine vs. six negative coefficients of COI, respectively; Figure 5). Therefore, the prediction on the spatial avoidance of human disturbance was confirmed for all species except the hare, the wild cat and the wolf.

### Temporal activity

**FIGURE 6:**
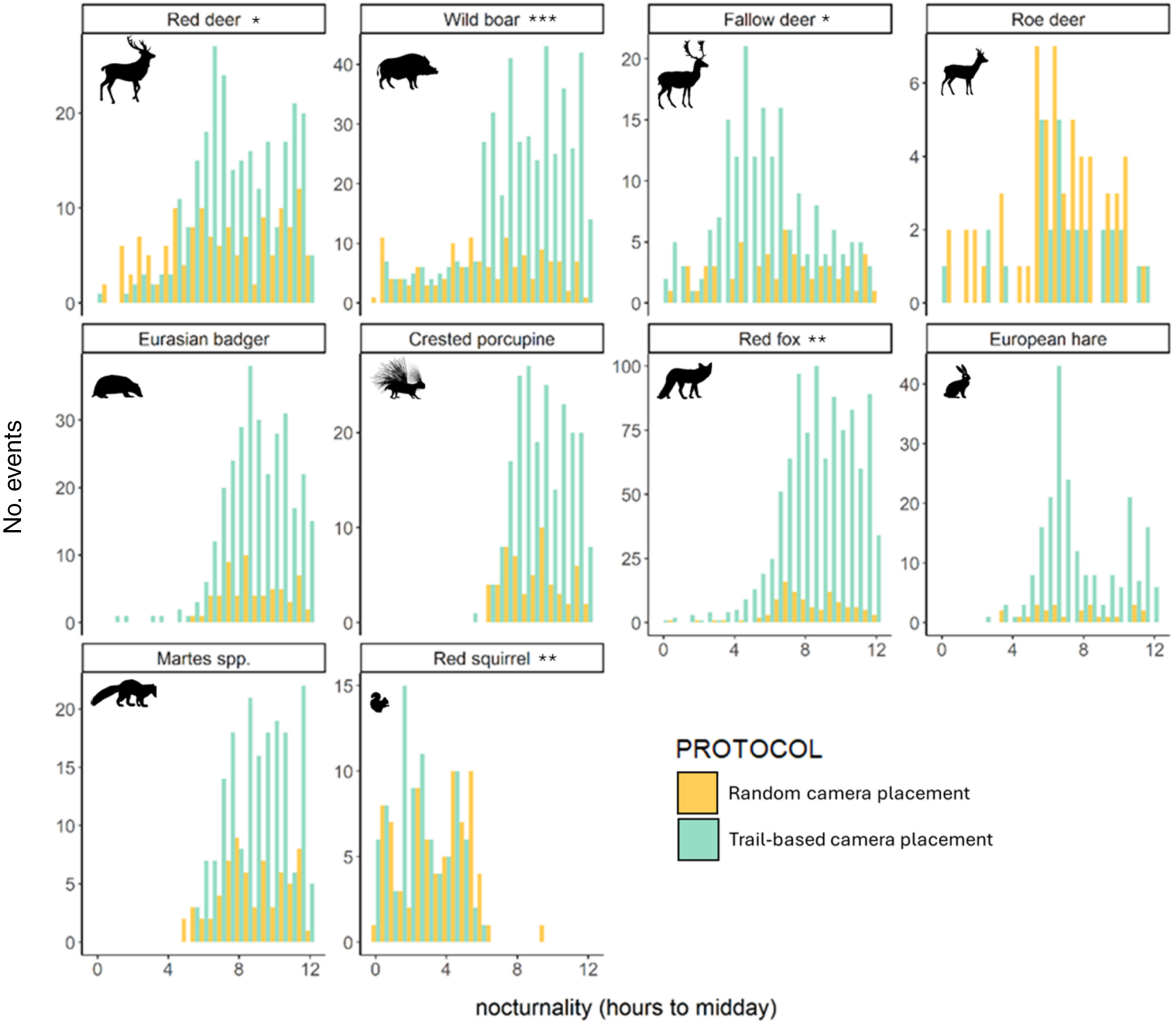
Barplots show the degree of nocturnality (*x* axis; expressed as hours from solar noon) of the 10 mammal species that had enough photographic events in both sampling designs. The *y* axis reports the absolute count of events for the off‐trails (yellow bars) and the on‐trails design (light blue bars). Asterisks represent significance level (*p < 0.05; **p < 0.01; ***p < 0.001). Wildlife silhouettes were downloaded from the public domain website Openclipart (https://openclipart.org/).

The diel activity pattern of almost all mammals considered did not differ significantly between on‐and off‐trail placements, except for wild boar (*w* = 11.67; *p* < 0.001) and fallow deer (*w* = 10.71; *p* < 0.01) that were more cathemeral off‐trails and crepuscular on‐trails (Appendix S1: Figure S3). All species generally had a high temporal overlap (Δ > 0.70) when comparing the diel activity curves between the two sampling designs. Nonetheless, the analysis on the degree of nocturnality revealed that wild boar (on‐trails = mean 8.20 ± 2.7 SD; off‐trails = 6.18 ± 3.3; *w* = 43,794, *p* < 0.001), red deer (on‐trails = 7.88 ± 2.5; off‐trails = 7.09 ± 3.2; *w* = 21,993, *p* < 0.05) and fox (on‐trails = 8.80 ± 2.0; off‐trails = 8.28 ± 2.1; *w* = 59,826.5, *p* < 0.01; Figure 6) had a higher nocturnality on‐ than off‐trails. Also, porcupine, *Martes* spp., badger, hare and roe deer tended to have higher average nocturnality values from sampling on‐trails, but the differences were not significant (Figure 6). Conversely, red squirrel (on‐trails = 2.54 ± 1.6; off‐trails = 3.4 ± 2; *w* = 2411, *p* < 0.01) and fallow deer (on‐trails = 5.71 ± 2.6, off‐trails = 6.73 ± 3.0; *w* = 4259.5, *p* < 0.05) were less nocturnal on‐ than off‐trails. Therefore, the prediction of higher nocturnality on trails was supported only for a subset of species.

## DISCUSSION

By simultaneously monitoring a mammalian community with off‐ and on‐trail systematic camera trapping at the same spatial and temporal scales, we obtained directly comparable data to assess benefits and drawbacks of the two sampling strategies for community studies. Our main findings were that: (1) both strategies were successful in sampling the target community, although the trail‐based design collected a markedly greater amount of data in terms of species richness and independent events, whereas the off‐trail design rarely detected carnivores; (2) the relationships between environmental variables and both mammal occupancy and site use obtained with the two designs were not completely equivalent, suggesting that a combination of both strategies would be more suited to study habitat use; (3) the effect of human visitation, concentrated on the trail network, penetrates through the surrounding habitat, causing spatial avoidance responses in many mammal species not only on highly frequented trails, but also on surrounding off‐trail sites; (4) temporal activity curves were generally similar between designs, although some species had a more marked nocturnal behavior from sampling on trails.

In agreement with Cusack et al. (2015) and Tanwar et al. (2021), we found that the trail‐based design resulted in a faster data accumulation and considerably greater photographic rate for most species, especially carnivores. Many carnivores are known to exploit linear features to minimize energy expenditure during movement and increase the probability of encountering prey (Dickie et al., 2017; Weckel et al., 2006). Furthermore, the likelihood of capturing rare and elusive species is generally greater along trails for their use as travel routes (Greco et al., 2023; Greco & Rovero, 2021). In line with these expectations, the data collected for wolf and wildcat off‐trails were insufficient for statistical modeling.

Oppositely to what was found by Blake and Mosquera (2014), Fonteyn et al. (2021) in tropical environments and Cusack et al. (2015) in arid environments, we found that community composition was clearly distinct between the two designs, with the relative abundance of carnivores that was almost double on‐ than off‐trails. Such a difference may be determined by the different habitats and related forest management strategies, with temperate forests in high‐income countries that probably have more extensive and widespread road and trail networks than, for example, tropical forests. Indeed, forestry roads are known to be largely used by large carnivores as a fast way to move between patches of the landscape, while avoiding humans at the fine scale (Zimmermann et al., 2014). Hence, the greater availability of forestry roads and hiking trails may exacerbate the differential use of linear features by mammals, leading to differences in community composition and photographic rates between sampling strategies. Though the proportion of relative abundances of carnivores and herbivores detected off‐trails is probably closer to real densities, off‐trail placement determines a lack of data for carnivores, with potential consequent biases on the study of predator–prey relationships. Harmsen et al. (2010) found that different species have distinct tendencies of using trails, leading to potential biases in their detection probability, thus relative abundance indexes, when monitoring with trail‐based designs. In our case, only the fallow deer and roe deer had the notable exception of higher detection probability off trails. These cervids might avoid trails to minimize encounters with predators and humans (Dickie et al., 2017) or may be more morphologically adapted to traverse dense understory vegetation and bushes. Nonetheless, the photographic rate and detection probability were still sufficiently high on trails to allow further analyses and ecological considerations on this species also when using the trail‐based sampling strategy.

When we modeled species responses to environmental and anthropogenic variables in terms of occupancy and site‐use intensity with the two sampling designs, we found that coefficients were concordant for 66% of the species‐variable combinations. However, only for 18% of these (equally represented by occupancy and site‐use intensity) the inference from the two strategies lead to the same conclusion in terms of statistical significance and ecological relevance of the effect. For example, fallow deer had significantly lower occupancy in relation to human frequentation with both sampling strategies (i.e., its probability of presence decreased both on highly frequented trails and adjacent off‐trail sites). Conversely, for 27% of cases, the use of one sampling design over the other would result in different ecological inferences since CIs were overlapping zero for only one design, and they had different effect sizes: for example, fallow deer occupancy on trails significantly increased as distance to the border of the protected area increased, whereas this effect was not significant with the off‐trail sampling. Therefore, when the assessment of species‐habitat relationships is the main study goal, a mixture of both sampling designs might be ideal to correctly gather broader information on mammals' space use, as already highlighted by Blake and Mosquera (2014), Di Bitetti et al. (2014), Hofmeester et al. (2021) and Iannarilli et al. (2021).

Mammals may use trails and the surrounding habitats during different behavioral phases, for example, exploiting trails for direct and faster movement bouts, exiting the trail for foraging and resting (Rowcliffe et al., 2014). Habitat use can indeed vary between different activity types, and environmental variables that represent important drivers of space use during foraging or resting may differ from those related to linear movements (Klappstein et al., 2023; Nicosia et al., 2017). Deploying cameras both along trails and at off‐trail locations can therefore allow a more comprehensive representation of different behaviors and consequently also of the drivers of animal use of space. More research is needed to set a more specific survey design to obtain a robust combination of these methods for habitat use assessment.

When the assessment of habitat use is not a priority goal, trail‐based sampling seems a reasonable choice for monitoring mammal communities. Camera‐traps on trails yielded sufficient data for elusive species (i.e., carnivores) and even for mammals that were more detected by cameras off trails (i.e., roe deer), suggesting a higher per‐effort efficiency of this design as previously observed (Kolowski & Forrester, 2017). Trail‐based sampling can additionally be more efficiently repeated over time to estimate trends (Tanwar et al., 2021), and the higher detection probabilities of many species would improve the precision of occupancy model estimates. Trail‐based monitoring has indeed been successfully applied to obtain multiyear trends at community and species level (Ahumada et al., 2013; Salvatori et al., 2023). However, the interpretation of occupancy for on‐ and off‐trail sampling may be critical, as the different detection probabilities along trails affect occupancy estimates.

In our case, occupancy was higher for the trail‐based design for most species (Appendix S1: Figure S2). In its original definition, that is, the probability that a site is used by a species, occupancy was developed for discontinuous habitats, and its interpretation in a continuous environment has been proven problematic, being inextricably linked to home‐range size and population density, and dependent on what is defined as a “site” (Efford & Dawson, 2012). Hence, for the trail‐based design, occupancy estimates might be better interpreted as the probability of site use during linear movements and can be inflated by the preferential use of trails by mammals to move from one area of the home range to another. Instead, occupancy is presumably closer to its original definition when monitoring with the off‐trail design, and is likely related to foraging and resting behaviors. These two metrics can in some cases be strongly dissimilar, as we found for the crested porcupine whose occupancy on trails was thrice that off trails. For these reasons, caution should be taken when interpreting occupancy estimates that are unlikely to be comparable across areas or studies that deployed different sampling designs. Additionally, it is important to remark that a trail‐based sampling is not suitable for density estimation studies of unmarked animals through random encounter and similar models that require random placement of camera‐traps (Palencia et al., 2021).

An inherent advantage of trail‐based sampling lies in the possibility of concomitantly collecting data on wildlife and potential sources of disturbance such as livestock (Greco et al., 2022; Salvatori et al., 2022) and humans at the same spatiotemporal resolution (Salvatori et al., 2023, 2024). However, human mobility data, with the rise of popular social‐media platforms for tracking outdoor activities, allows us to indirectly quantify the effects of human disturbance even when direct information is not available (Corradini et al., 2021). Hence, the use of COI enabled us to understand the effect of human recreation also outside the trail network, thus comparing information from the two designs. Our results showed that the site use of most target species decreased as proximity to heavily used human trails increased. Evidence indicates that mammals adjust their spatiotemporal use of trails to avoid humans, and that body mass, hunting status, and habitat structure play a role (Nickel et al., 2020; Salvatori et al., 2024). Yet, the effect that human disturbance concentrated on trails exerts outside of the trail network is less studied, with available evidence reporting spatial avoidance of disturbance in the vicinities of trails (Pépin et al., 1996; Westekemper et al., 2018).

The spatial avoidance of sites closer to more intensively used hiking trails and forestry roads that we detected could have important consequences in terms of aggregation of animals in areas with lower rates of human recreation, with cascading outcomes on forest browsing and soil nutrient cycling (Di Nicola et al., 2023; Segar et al., 2022). Although anthropogenic linear features such as hiking trails and unpaved roads may cover an overall small proportion of a protected area, their ecological effect can be much wider, affecting a considerable extent of the available habitat for wild mammals and potentially altering interspecific interactions (DeMars & Boutin, 2018; Seigle‐Ferrand et al., 2022). Our results delineate anthropogenic disturbance as a variable that has repercussions beyond the linear elements where it is concentrated, with potential consequences across taxa (Suraci et al., 2019).

Being concentrated during the central hours of the day, human presence has a clear temporal component that elicits avoidance in mammals through the modulation of their activity pattern (Gaynor et al., 2018). This behavioral strategy dynamically responds to varying degrees of human disturbance along trails, for example, by limiting diurnal and crepuscular activity in more disturbed sites (Salvatori et al., 2024). Here, for wild boar, red deer, and red fox, we recorded a greater nocturnal behavior from the on‐ than off‐trail design. However, the temporal activity curves obtained with the two designs were generally similar for most species, with high overlap coefficients. Hence, at least in our study case, the use of one design over the other would not lead to important biases in the estimation of mammals' temporal patterns. The opposite was found in Tanwar et al. (2021), for which mammals' temporal patterns differed on trails compared to off‐trails. However, the overlap coefficients were still moderately high for four over five species considered (Δ > 0.50).

In conclusion, we show that camera‐trap placement has profound implications on the results of mammalian studies and should not, therefore, be overlooked when planning new studies. Current efforts on data harmonization are favoring camera‐traps installations along trails for large‐scale and multispecies research on mammals (Cove et al., 2021; Greco et al., 2025; Kays et al., 2022; Shamon et al., 2024). In this regard, we found that the sampling design with camera‐traps systematically located along trails and forestry roads was more effort‐effective for monitoring the community of medium‐to‐large mammals through a set of commonly used metrics. We acknowledge that our results primarily refer to a setting of dense mountainous temperate forests. However, the greater photographic rate of mammals and the possibility to collect sufficient data for carnivores match results from comparable studies conducted in semiarid environments, characterized by a matrix of savannah and woodlands (Cusack et al., 2015; Tanwar et al., 2021), hence extending the validity of our findings to more open, wooded habitats. Additionally, trail‐based designs can also record passing humans and livestock, providing for direct inference on the effect of anthropogenic activity on wildlife, though human mobility data can help in obtaining information on human frequentation even when sampling outside the trail network. Lastly, our results corroborate previous findings that a mixed approach with cameras located both on‐ and off‐trails would be best suited to study habitat use of mammals.

## CONFLICT OF INTEREST STATEMENT

The authors declare no conflicts of interest.

## Supporting information


Appendix S1.

## Data Availability

Data (Greco, 2025) are available in Figshare at https://doi.org/10.6084/m9.figshare.29423327.v1.
