# Supplementary material for: Placement matters: Implications of trail‐ versus random‐based camera‐trap deployment for monitoring mammal communities
Source: Ecol Appl. 2025 Aug 7;35(5):e70083. doi: 10.1002/eap.70083 (PMC12329709; doi:10.1002/eap.70083)
Supplement: Supplementary file 1 — Appendix S1. [file EAP-35-e70083-s001.pdf]

Appendix S1

Ecological Applications

Placement matters: Implications of trail- versus random-based camera-trap deployment for monitoring mammal communities

Ilaria Greco, Marco Salvatori, Elena Buonafede, Alessandra Pistolesi, Andrea Corradini, Nadia Cappai, Matilde Marconi, Lorenzo Seidenari, Francesca Cagnacci and Francesco Rovero

SUPPLEMENTARY TEXT

Specifications of the Multi-Species Occupancy Model and GLMs on site-use intensity

For each sampling protocol, detection/non-detection data were organised into a species by sites matrix. MSOMs are based on the encounter histories of species *i* across sites *j* within an area, during multiple sampling occasions *k*. True occurrence (i.e., presence/absence) is a state variable defined as $z_{ij} = 0$ when species *i* is not present at site *j*, and $z_{ij} = 1$ when it is present. Due to imperfect detection (i.e., $p < 1$), true occurrence is latent and modelled with a Bernoulli distribution $z_{ij} \sim Bern\ (\psi_{ij})$, where ψ_{ij} is the site-specific occupancy probability of each species. Detection frequences in terms of number of days species *i* was recorded at site *j* are modelled as a Binomial random variable $y_{ijk} \sim Binom\ (p_{ij}\ z_{ij},\ k)$, where p_{ij} is the detection probability of species *i* to be detected at site *j* when $z_{ij} = 1$ and *k* is the total number of sampling occasions (i.e., number of days the CT was active).

Detection and occupancy probabilities can be modelled as a function of covariates with a logit-link function (Kéry & Royle, 2015). For the random-base design, we modelled *p* in relation to the distance from the camera-trap to the closest municipality (*DIST_TOWN*), calculated as the Euclidean distance from each sampling point to the centroid of the closest town; and to the mass of each species expressed in logarithmic scale (*LOGMASS*), extracted from Smith et al. (2003), to correct for the potentially higher detection of larger-sized species. For the trail-based design, besides the same covariates used for the random design, we also included the categorical variable *ROAD_TRAIL*, that indicated whether the sampling site was located on forestry roads or hiking trails:

$$\text{logit}(p_{ij})_{\text{random}} = \theta R + \alpha 1R_i * \text{DIST\_ } TOWN_{\text{j}} + \alpha 2R * \text{LOGMASS}$$

$$\text{logit}(p_{ij})_{\text{trail}} = \theta T + \alpha 1T_i * \text{DIST\_ } TOWN_{\text{j}} + \alpha 2T\ \text{LOGMASS} + \alpha 3T_i * \text{equals}(\text{ROAD\_TRAIL}_{\text{j}},2) + \alpha 4T_i * \text{equals}(\text{ROAD\_TRAIL}_{\text{j}},3)$$

Where $\alpha 1R_i$, $\alpha 1T_i$, $\alpha 3T_i$ and $\alpha 4T_i$ are species-specific coefficients.

For the occupancy modelling, we extracted elevation (*ELEV*) and terrain slope (*SLOPE*) from Tinitaly (Tarquini et al., 2023), a Digital Elevation Model with 10 m resolution, and calculated the Euclidean distance of each sampling site to the closest municipality (DIST_ TOWN) and to the protected area border (DIST_BORDER; IUCN & UNEP-WCMC, 2020). To include information on anthropogenic disturbance from outdoor activities we extracted the Cumulated Outdoor activity Index COI (Corradini et al., 2021). The index is calculated from the human mobility data cumulated over one year from the freely viewable Strava heatmap (https://www.strava.com/maps/global-heatmap), an application developed by STRAVA (San Francisco, CA, USA), a popular smartphone application used by recreationists to track outdoor activities. The detailed procedure to extract the COI is reported in Corradini et al. (2021). For this study, we extracted the heatmap with all cumulated activity types (on-foot, cycling, etc.) recorded over the course of 12 months prior to May 2023 (when the extraction was performed) at a zoom level of 11, for a final raster of 76 m resolution. We thus derived a density of disturbance by summing the values of the raster cells in a 300 m circular buffer for each sampling site in both sampling designs.

$$\text{logit}(\psi_{ij})_{\text{random}} = \eta R + \beta 1\text{R}_i * \text{ELEV}_j + \beta 2\text{R}_i * \text{SLOPE}_j + \beta 3\text{R}_i * \text{DIST\_ TOWN}_j + \beta 4\text{R}_i * \text{DIST\_BORDER}_{\text{j}} + \beta 5\text{R}_i * \text{COI}_{\text{j}}$$

$$\text{logit}(\psi_{ij})_{\text{trail}} = \eta T + \beta 1\text{T}_i * \text{ELEV}_j + \beta 2\text{T}_i * \text{SLOPE}_j + \beta 3\text{T}_i * \text{DIST\_ TOWN}_j + \beta 4\text{T}_i * \text{DIST\_BORDER}_{\text{j}} + \beta 5\text{T}_i * \text{COI}_{\text{j}}$$

Coefficients $\beta 1\text{R}_i - \beta 5\text{R}_i$ and $\beta 1\text{T}_i - \beta 1\text{R}_i$ are also species-specific coefficients.

Given the binary nature of occupancy that considers only detection/non-detection data and does not discriminate well between sites visited more frequently and those used rarely, quickly reaching an asymptote towards ψ =1 (Salvatori et al., 2023), we decided to model also the number of events as a proxy of site-use

intensity. To do so, we fitted for each sampling design a GLM with Poisson distributions, including the same set of covariates used for the occupancy modelling above. The GLMs had the following structure:

$$event\_random_{ij} \sim Poisson\ (\lambda event\_random_{ij})$$

$$event\_trail_{ij} \sim Poisson\ (\lambda event\_trail_{ij})$$

where $event\_random_{ij}$ and $event\_trail_{ij}$ represent the site- and species-specific number of events, modelled as a function of covariates through a log link function:

$$\log(\lambda event\_random_{ij}) = \eta E_{ran} + \beta E1_{ran} * ELEV_j + \beta E2_{ran} * SLOPE_j + \beta E3_{ran} * DIST\_TOWN_j + \beta E4_{ran} * DIST\_BORDER_j + \beta E5_{ran} * COI_j + \log(CAM\_DAYS_j) + \varepsilon_{ij}$$

$$\log(\lambda event\_trail_{ij}) = \eta E_{trail} + \beta E1_{trail} * ELEV_j + \beta E2_{trail} * SLOPE_j + \beta E3_{trail} * DIST\_TOWN_j + \beta E4_{trail} * DIST\_BORDER_j + \beta E5_{trail} * COI_j + \log(CAM\_DAYS_j) + \varepsilon_{ij}$$

where ε_{ij} is a species- and sites-specific overdispersion term derived from a normal distribution, such that $\varepsilon_{ij} \sim Normal(0, \sigma^2_{ij})$. For every GLM, we used the log of the site-specific number of camera days (CAM_DAYS_j) as an offset to account for sampling effort.

All covariates considered were not collinear (Pearson's < 0.60 as threshold) and were scaled to have mean 0 and unit standard deviation. We fitted the models in a Bayesian framework by using the package *jagsUI* (Kellner & Meredith, 2021) from R (R Core Team, 2022). We used Markov Chain Monte Carlo, with 3 chains and 375,000 iterations for each model (125,000 adaptation and 250,000 burn-in), and with a thinning value of 10. Chain convergence was verified both visually and by using the R-hat diagnostic, with R-hat ≈ 1.01 considered a successful convergence. We performed a Goodness-of-fit test on the residuals from the site-use intensity GLMs to check if the statistical assumptions of the models were met. To evaluate potential differences in species-specific detections and occupancy probabilities, we compared post-Hoc the value of detection and occupancy estimated by the MSOMs for the two monitoring protocols with Wilcoxon signed rank tests.

References

Corradini, A., Randles, M., Pedrotti, L., van Loon, E., Passoni, G., Oberosler, V., Rovero, F., Tattoni, C., Ciolli, M., & Cagnacci, F. (2021). Effects of cumulated outdoor activity on wildlife habitat use. Biological Conservation, 253, 108818. https://doi.org/10.1016/j.biocon.2020.108818

Kellner, K., & Meredith, M. (2021). Package 'jagsUI'. A Wrapper Around "rjags" to Streamline "JAGS" Analyses. R package. https://cran.r-project.org/web/packages/jagsUI/jagsUI.pdf

Kéry, M., & Royle, J. A. (2015). Applied Hierarchical Modeling in Ecology: Analysis of distribution, abundance and species richness in R and BUGS - 1st Edition. https://shop.elsevier.com/books/applied-hierarchical-modeling-in-ecology-analysis-of-distribution-abundance-and-species-richness-in-r-and-bugs/kery/978-0-12-801378-6

R Core Team. (2022). R: A language and environment for statistical computing. https://www.R-project.org/

Salvatori, M., Oberosler, V., Rinaldi, M., Franceschini, A., Truschi, S., Pedrini, P., & Rovero, F. (2023). Crowded mountains: Long-term effects of human outdoor recreation on a community of wild mammals monitored with systematic camera trapping. Ambio. https://doi.org/10.1007/s13280-022-01825-w

Smith, F. A., Lyons, S. K., Ernest, S. K. M., Jones, K. E., Kaufman, D. M., Dayan, T., Marquet, P. A., Brown, J. H., & Haskell, J. P. (2003). Body Mass of Late Quaternary Mammals. Ecology, 84(12), 3403–3403. https://doi.org/10.1890/02-9003

Tarquini, S., Isola, I., Favalli, M., Battistini, A., & Dotta, G. (2023). INITALY, a digital elevation model of Italy with a 10 meters cell size (Version 1.1). Istituto Nazionale di Geofisica e Vulcanologia (INGV). https://doi.org/10.13127/tinitaly/1.1

Table S1. Overview on the relevant literature that compared different types of camera-trap sampling designs, with main outcomes and recommendations.

Study	Placements compared	Main findings	Recommendations
Di Bitetti et al., 2014	trails vs. off-trails	Higher recording rate and species richness on than off roads and dissimilar mammal assemblage.	Combination of trails and off-trails placement for a comprehensive description of mammals' abundance and composition.
Blake & Mosquera, 2014	trails vs. off-trails	Higher species richness off trails, rare species only captured on trails. Similar species accumulation and community composition.	Combination of trails and off-trails placement for a comprehensive description of mammals' abundance and composition.
Cusack et al., 2015	Random vs. game-trails	Marginal differences in richness and composition. Trail-based required lower sampling effort. Carnivores and large-body mammals caught on trails.	The type of sampling design does not affect community-level inference when effort is adequate, yet random placement is recommended.
Kolowski & Forrester, 2017	Random vs. game-trails or log features	Capture rate, species richness and detection probability were higher at feature-based sites.	Random placement is recommended.
Fonteyn et al., 2021	Random vs. trails	Similar richness and composition. Similar detection probability and trapping rate. Similar species composition.	Trail-based placement is recommended to keep consistency with recent large-scale studies.
Hofmeester et al., 2021	Random vs. lynx-targeted	Lynx-targeted sites had highest detection probability for almost all species. Adding extra CTs increase detections.	Combination of lynx-targeted and random.
Iannarilli et al., 2021	Random vs. lured vs. trail-based	Species-specific differences. No evidence that feature-based sites have higher capture rate of carnivores.	Mixed survey-design strategy is recommended.
Tanwar et al., 2021	Random vs. trails	Similar species richness but faster accumulation rate with trail-based sites. Greater Relative abundance of carnivores on trails. Comparable group size. Different temporal activities.	Trail-based placement is supported although outcomes may be biased for herbivores.

Table S2. Naïve occupancy (ψ) and number independent detection events (>30 min. between events) of mammal species detected with the random and the trail-based camera-trapping design.

Wild species			RANDOM DESIGN		TRAIL-BASED DESIGN	
**Order**	**Species**	**Common name**	**Naïve ψ**	**Events**	**Naïve ψ**	**Events**
Carnivora	*Canis lupus*	Wolf	0.07	5	0.76	242
	Felis silvestris	Wild cat	0.07	5	0.53	80
	Martes sp.	Martesd species	0.46	68	0.69	182
	Meles meles	Eurasian badger	0.44	63	0.81	303
	Mustela potorius	European polecat	0.15	9	0.2	16
	Procyon lotor	Raccon	-	-	0.03	2
	Vulpes vulpes	Red fox	0.51	107	1	962
Erinaceomorpha	*Erinaceus europaeus*	European hedgehog	0.02	1	0.02	2
Lagomorpha	*Lepus europaeus*	European hare	0.15	26	0.53	213
Rodentia	*Glis glis*	European edible dormouse	0.02	6	0.02	1
	Hystrix cristata	Crested porcupine	0.24	54	0.69	211
	Sciurus vulgaris	Eurasian red squirrel	0.29	78	0.24	83
Ungulata	*Capreolus capreolus*	European roe deer	0.39	58	0.32	30
	Cervus elaphus	Red deer	0.68	141	0.71	255
	Dama dama	Fallow deer	0.24	56	0.39	182
	Sus scrofa	Wild boar	0.63	144	0.9	441
-	-	Small mammals	0.46	121	0.25	88
Domestic, humans & vehicles						
Artiodactyla	*Bos taurus*	Domestic cattle	-	-	0.15	84
Carnivora	*Canis lupus familiaris*	Domestic dogs	0.07	4	0.59	358
	Canis lupus familiaris	Feral dogs	0.02	1	0.22	17
	Felis catus	Domestic cats	0.07	8	0.08	29
Perissodactyla	*Equus asirus*	Domestic donkey	-	-	0.02	2
	Equus ferus caballus	Domestic horse	-	-	0.15	56
Primates	*Homo sapiens*	Human	0.54	70	1	1872
-	-	Vehicle	-	-	0.58	574

Table S3. Summary table for the detection probabilities derived from the multi-species occupancy models from the two sampling designs (i.e., trail-based and random). The table shows the species-specific mean of the posterior distribution estimated for every variable, the standard deviation, the 90% Bayesian Credible Interval, the potential scale reduction statistics (R-hat) which indicates chains convergence when values are close to 1, and the number of samplings from the posterior distribution (n.eff).

Sub-model	Variable	Species	Mean	SD	5.00%	95.00%	R-hat	n.eff
Detection probability (p) Trail-based design	Dist. Town	Crested porcupine	0.05	0.10	-0.12	0.21	1	37500
		Eurasian badger	0.01	0.08	-0.12	0.13	1	37500
		Eurasian red squirrel	-0.48	0.14	-0.71	-0.25	1	37500
		European hare	-0.05	0.13	-0.27	0.17	1	37500
		European polecat	0.35	0.37	-0.24	0.98	1	4960
		European roe deer	-0.13	0.25	-0.54	0.28	1	37500
		Grey wolf	-0.06	0.08	-0.20	0.07	1	16836
		Martes species	-0.12	0.09	-0.27	0.03	1	37500
		Persian fallow deer	0.06	0.14	-0.18	0.29	1	30114
		Red deer	0.25	0.09	0.10	0.40	1	16845
		Red fox	-0.18	0.06	-0.27	-0.08	1	37500
		Wild boar	0.09	0.07	-0.03	0.22	1	37500
		Wild cat	0.21	0.13	-0.01	0.43	1	9617
	Camera on Trails	Crested porcupine	0.02	2.25	-3.67	3.74	1	13227
		Eurasian badger	0.03	2.25	-3.69	3.75	1	25743
		Eurasian red squirrel	-0.03	2.25	-3.70	3.69	1	25391
		European hare	0.00	2.25	-3.68	3.69	1	37500
		European polecat	0.00	2.25	-3.70	3.69	1	17545
		European roe deer	0.00	2.26	-3.72	3.72	1	23938
		Grey wolf	0.00	2.26	-3.70	3.73	1	37500
		Martes species	0.00	2.25	-3.68	3.70	1	36374
		Persian fallow deer	0.01	2.25	-3.67	3.71	1	37500
		Red deer	-0.01	2.26	-3.75	3.70	1	37500
		Red fox	0.00	2.26	-3.68	3.74	1	37500
		Wild boar	-0.01	2.26	-3.71	3.69	1	37500
		Wild cat	-0.02	2.25	-3.72	3.67	1	36464
	Camera on Roads	Crested porcupine	0.00	0.23	-0.39	0.38	1	32040
		Eurasian badger	-0.19	0.18	-0.49	0.11	1	37500
		Eurasian red squirrel	-0.87	0.61	-1.94	0.06	1	37500
		European hare	0.26	0.21	-0.08	0.61	1	33997
		European polecat	-0.77	1.01	-2.57	0.72	1	37500
		European roe deer	-0.43	0.48	-1.25	0.32	1	18289
		Grey wolf	0.38	0.17	0.10	0.66	1	37500
		Martes species	-0.66	0.25	-1.08	-0.26	1	37500
		Persian fallow deer	-0.20	0.36	-0.80	0.37	1	17398
		Red deer	-0.42	0.21	-0.77	-0.08	1	21308
		Red fox	0.17	0.12	-0.03	0.38	1	37500
		Wild boar	-0.11	0.16	-0.37	0.15	1	14214
		Wild cat	-0.18	0.31	-0.70	0.32	1	20556
	Log	not species-specific	-0.02	0.21	-0.39	0.30	1.01	112
Detection probability (p) Random design	Dist. Town	Crested porcupine	0.07	0.26	-0.37	0.5	1	14483
		Eurasian badger	0.09	0.13	-0.13	0.31	1	37500
		Eurasian red squirrel	-0.3	0.16	-0.57	-0.03	1	37500
		European hare	-0.4	0.27	-0.85	0.05	1	24063
		European polecat	0.03	0.27	-0.41	0.46	1	27087
		European roe deer	0.33	0.19	0.03	0.65	1	37500
		Martes species	0.15	0.13	-0.06	0.36	1	37500
		Persian fallow deer	0.27	0.16	0.01	0.54	1	37500
		Red deer	-0.2	0.1	-0.37	-0.03	1	37500
		Red fox	-0.11	0.12	-0.31	0.08	1	27972
		Wild boar	0.05	0.09	-0.1	0.21	1	37500
	Log	not species-specific	0.1	0.11	-0.08	0.1	0.26	1569

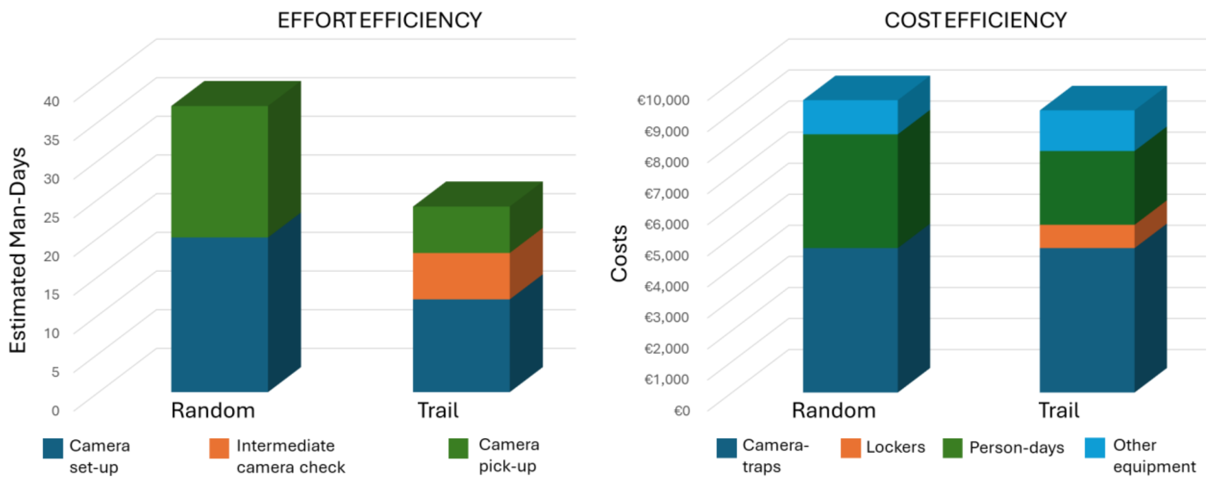

Figure S1. On the left, comparison of the effort efficiency expressed as number of man-days between a random- and trail-based camera-trapping monitoring strategy. On the right, comparison of the presumed costs needed for random-based and trail-based camera-trap projects. Costs include the prices of Browning Dark Ops camera-traps, lockers, other equipment (i.e., SD cards, batteries, battery chargers) and person-days. This latter was estimated as the daily salary multiplied by working days of a regular research fellow.

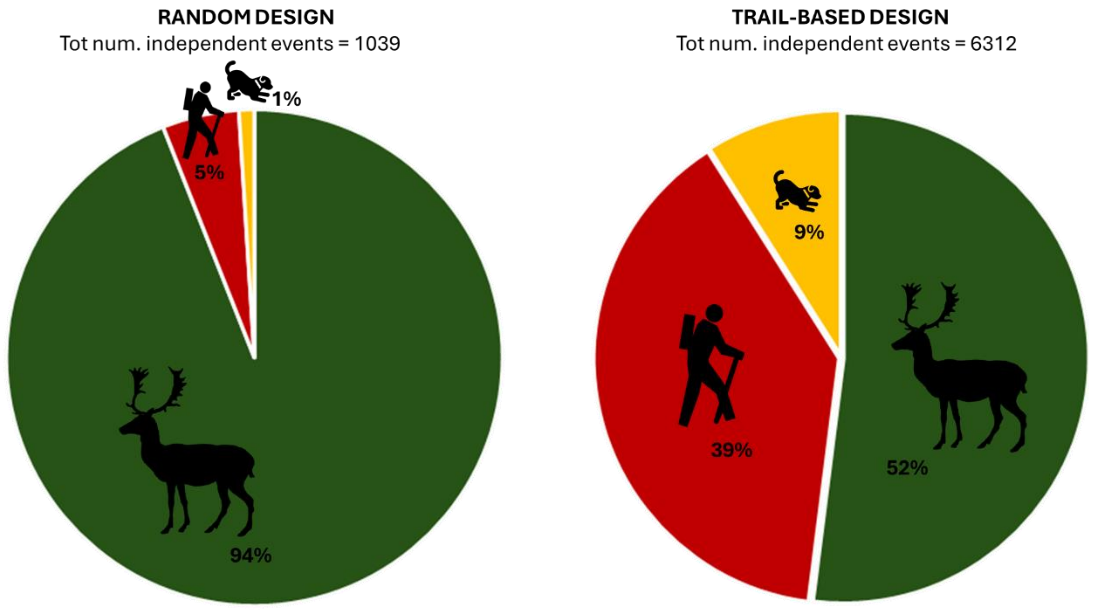

Figure S2. Percentage of independent events derived from a random and a trail-based camera-trapping design, divided into three main categories: wild mammals (green), humans (red) and domestic animals (yellow). Wildlife silhouettes were downloaded from the public domain website Openclipart (https://openclipart.org/).

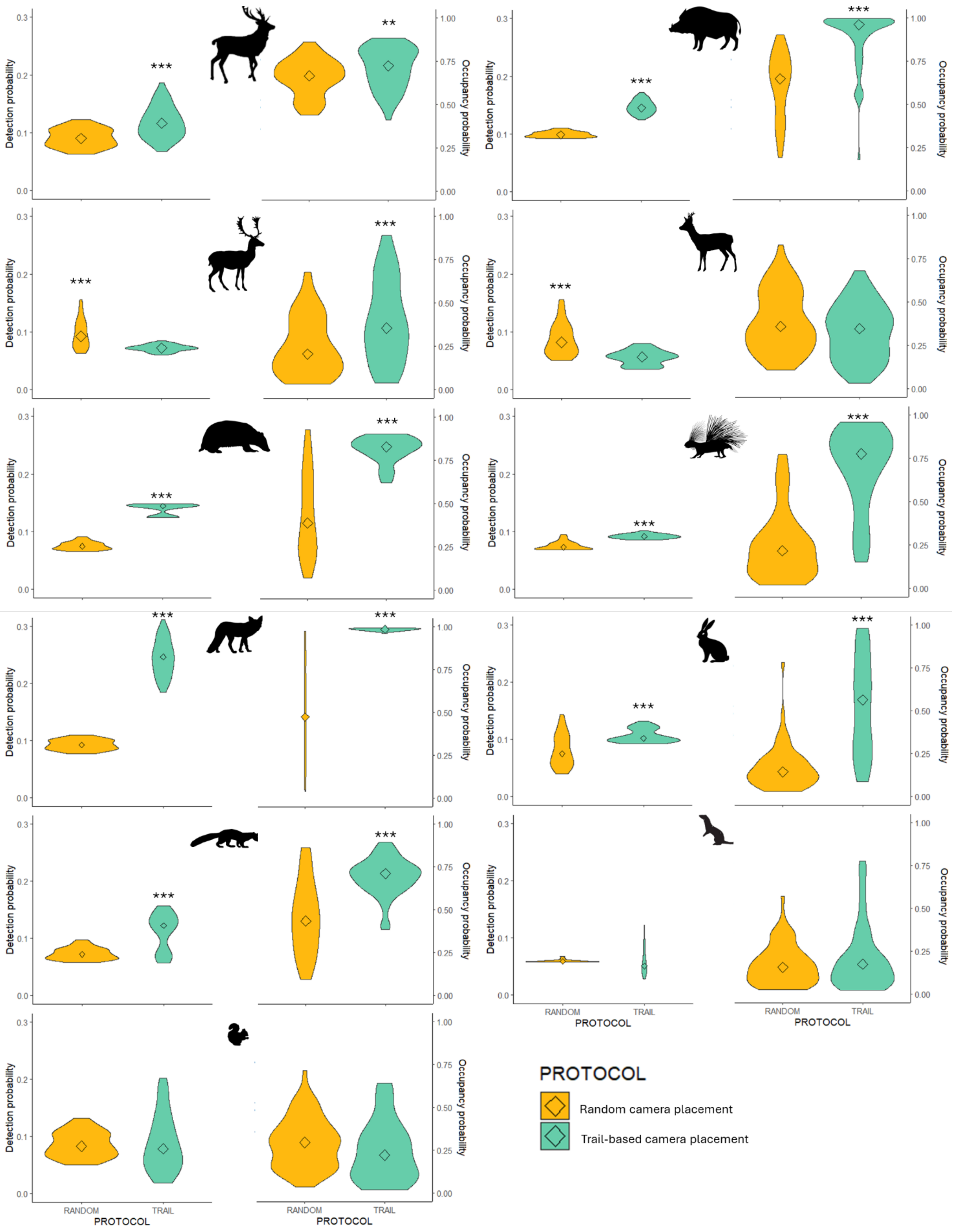

Figure S3. Species-specific violin plots for the comparisons of detections and occupancy probabilities estimated with multi-species occupancy models for a random stratified camera-trapping sampling design (yellow) and a trail-based camera-trapping protocol (green). Asterisks indicate significant differences estimated with a pairwise Wilcoxon signed rank test (. P > 0.09; * P < 0.05; ** P 0.01; P < 0.001). Wildlife silhouettes were downloaded from the public domain website Openclipart (https://openclipart.org/).

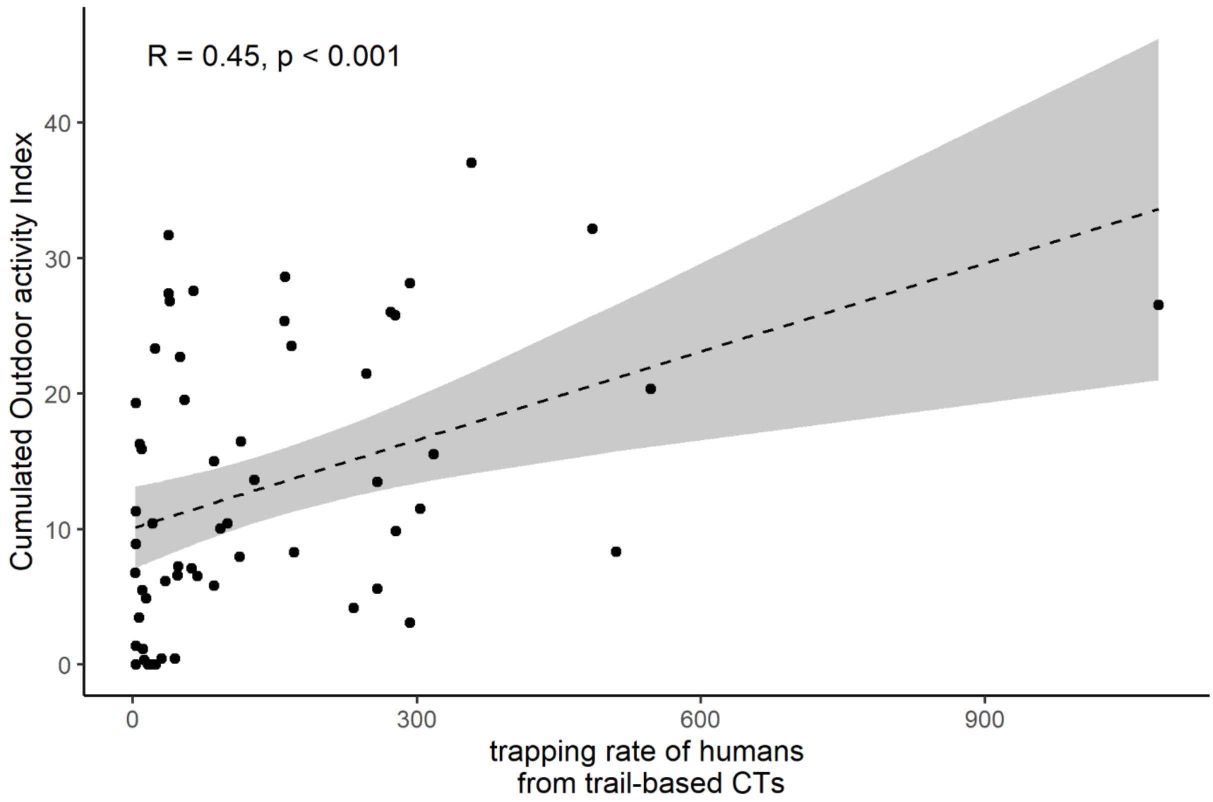

Figure S4. Correlation between the Cumulative Outdoor activity Index summed over a circular buffer of 300 m from each sampling site and trapping rate of humans from trail-based CTs (expressed as number of independent detection events over sampling days multiplied by 100). The regression line (dashed line with 95% confidence interval) is represented along the raw data (dots).

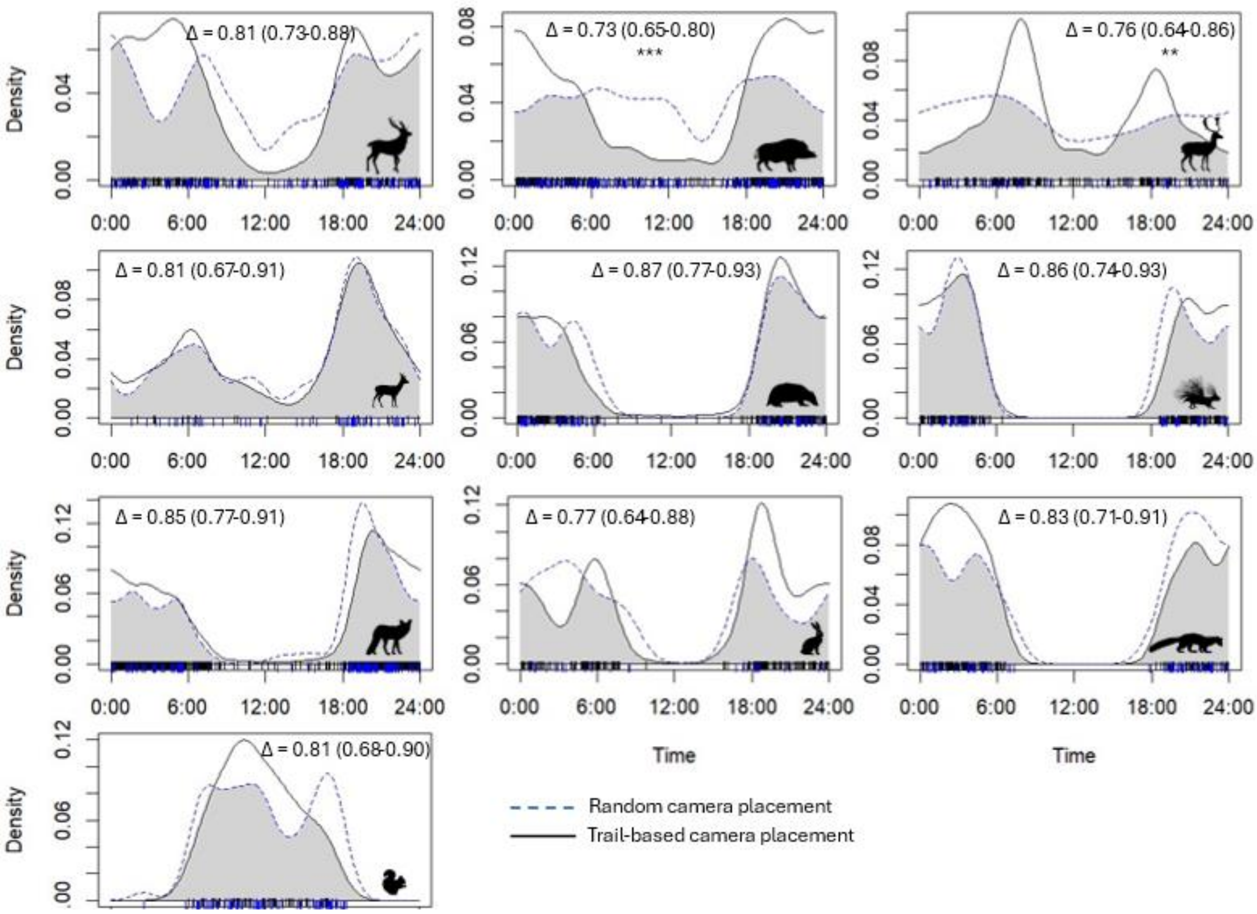

Figure S5. Comparison between species-specific activity pattern curves derived from a stratified random (dotted blue line) and a trail-based (solid black) sampling design. Species are ordered according to decreasing body mass. Only species with > 5 independent detection events have been included. Asterisks indicate significant differences estimated with Wald test (* P < 0.05; ** P 0.01; P < 0.001). Wildlife silhouettes were downloaded from the public domain website Openclipart (https://openclipart.org/).
